# Associations of insulin-like growth factor 1 and IGF binding proteins 2 and 3 with lipids in toddlers

**DOI:** 10.1186/s12944-026-02872-y

**Published:** 2026-02-03

**Authors:** Johanna Neiß, Hans Demmelmair, Fabian Geyer, Alexander Triebswetter, Dudung Angkasa, Joaquin Escribano, Natalia Ferré, Mariona Gispert-Llauradó, Berthold Koletzko, Susanne Bechtold-Dalla Pozza, Veit Grote

**Affiliations:** 1 Division of Metabolic and Nutritional Medicine, Department of Paediatrics, Dr. Von Hauner Children´s Hospital, LMU University Hospital, Munich, Germany; 2 German Center for Child and Adolescent Health, Munich, Germany; 3 Paediatrics Research Unit, Universitat Rovira I Virgili-IISPV, Reus, 43201 Spain; 4 Integriertes Sozialpädiatrisches Zentrum im Dr. Von Haunerschen Kinderspital, Munich, Germany

**Keywords:** IGF-1, Lipids, Toddlers, LDL cholesterol, IGF binding proteins

## Abstract

**Background and objectives:**

The insulin-like growth factor (IGF) axis is postulated to influence early-life adipocyte activity and lipolysis. We investigated whether IGF-1 and IGF-binding proteins (IGFBP-2, IGFBP-3) are associated with the lipids of healthy toddlers.

**Methods:**

Data were collected during the Toddler Milk Intervention trial to test the effect of milk protein on growth in the second year of life in Germany and Spain. Any blood values for IGF-1, IGFBP-2, IGFBP-3, low- and high-density lipoprotein cholesterol (LDL-C, HDL-C), total cholesterol (TC) and triglycerides (TG) were available at 12 and 24 months from 881 to 775 toddlers, respectively. These blood parameters were available from 444 children at both time points. Mixed intercept linear models adjusted for sex, fasting duration, country, and body mass index (BMI) were used to assess the associations between the IGF axis and lipids.

**Results:**

BMI was 17.0 ± 1.4 kg/m^2^ (M ± SD) at 12 months. IGF-1, IGFBP-3 and IGFBP-2 levels were 75.9 ± 36.1 ng/ml, 2630.3 ± 638.4 ng/ml and 575.5 ± 245.6 ng/ml, respectively; the mean LDL-C, HDL-C, TC and TG levels were 80.5 ± 22.9 mg/dl, 42.2 ± 10.9 mg/dl, 141.7 ± 25.5 mg/dl and 101.2 ± 58.9 mg/dl, respectively. While the IGFBP-2 and TG levels were higher at 12 months (*p* < 0.001) compared to 24 months, all other parameters were lower (*p* < 0.05). IGF-1 and IGFBP-3 were weakly positively associated with LDL-C, HDL-C and TC, whereas IGFBP-2 was weakly negatively associated with LDL-C, HDL-C and TC.

**Conclusion:**

The IGF axis is weakly associated with circulating lipids. IGF-1 and its binding proteins seem to have a limited impact on lipid profiles of toddlers.

**Trial registration:**

Clinical trial registration number: NCT02907502, 26.04.2016.

**Supplementary Information:**

The online version contains supplementary material available at 10.1186/s12944-026-02872-y.

## Introduction

Insulin-like growth factor (IGF) 1, growth hormone (GH), and insulin form an axis that signals the nutritional status of the organism to the cells so that they can grow, differentiate or undergo apoptosis [1]. The structural and functional properties of IGF-1 are similar to those of insulin [2]. Nutritional intake, especially the source and amount of protein, plays a role in regulating IGF-1 levels in young children. In general, breastfed infants have lower IGF-1 levels than formula-fed infants [3, 4]; it has also been shown that a lower protein content of infant formula can lead to lower IGF-1 levels [5]. Furthermore, cord blood IGF-1 was positively correlated with birthweight [6] and with gestational age [7]. While a lower birthweight is correlated with higher IGF-1 values in childhood [8].

In adults and children growth hormone deficiency has been shown to cause dyslipidemia [9]. IGF-I has been speculated to enhance free fatty acid utilization by muscle and to affect the flux of fatty acids into the liver [10]. In an in-vitro trial using fish cells, GH induced lipolysis during fasting, whereas IGF-1 inhibited this effect [11]. An adipogenic effect of IGF-1 was also found in rat- [12], pig- [13], and rabbit- [14]adipocyte precursor cells. The association of IGF-1 with lipids in adults presents a heterogeneous picture [15–17].

Real-life data showing that the IGF-1 axis is associated with lipid levels are scarce, especially in children. IGF-1 was positively associated with high-density lipoprotein cholesterol (HDL-C) and total cholesterol (TC) and negatively associated with triglycerides (TG) at birth in a study cohort including small for gestational age (SGA)-born infants [18]. In school-aged children, IGF-1 was positively associated with HDL-C [19, 20] but negatively associated with low-density lipoprotein cholesterol (LDL-C) [21, 22].

The bioavailability of IGF-1 is modulated by its binding proteins (IGFBPs), especially IGFBP-3 [23, 24]. IGFBP-3 has been shown to be positively associated with LDL-C, TC and TG [25]. In contrast, IGFBP-2 is negatively associated with metabolic syndrome risk [26] and the cardiovascular risk markers TC and TG [27].

About 20% of pediatric patients already have one or more elevated lipid parameters and approximately [28] 50% of children with lipid levels in the upper quartile have elevated lipid levels as adults [29]. This finding highlights the importance of screening children at increased risk for elevated lipid profiles to monitor their lipid levels early in life, if necessary.

We investigated the associations of IGF-1, IGFBP-2 and IGFBP-3 with LDL-C, HDL-C, TC, and TG in healthy toddlers during their second year of life. Based on the existing literature, we expected positive associations of IGF-1 with HDL-C and TC, and negative associations with LDL-C and TG. For IGFBP-3, we expected positive associations with all examined lipids, whereas for IGFBP-2 negative associations.

## Methods

### Study design

Blood was obtained within the Toddler Milk Intervention (ToMI) trial, which focused on the effects of milk protein in the second year of life on growth. The study was performed with healthy, term-born toddlers enrolled at 12 months of age in Germany (Munich) and Spain (Tarragona/Reus). The toddlers were assigned to receive one of two isocaloric young child formulas, containing a higher (6.1 g protein/100 kcal, comparable to the protein content of standard cows’ milk) or a lower (1.5 g /100 kcal, comparable to the protein content of breast milk in advanced lactation) amount of protein over the second year of life. The difference in protein content was calorically compensated with respective higher or lower fat content. The enrollment started in September 2016 and was finished in 2019. Further detailed information is provided in Grote et al. [30].

### Blood markers

Blood samples were taken at the 12-month (before intervention) and 24-month (after intervention) visits in plain and EDTA containing tubes, and serum and plasma samples were obtained via refrigerated centrifugation (10 min, 1500 g) and stored at -80 °C if not analyzed fresh. The levels of the blood lipids HDL-C, TC and TG were determined on the day of collection via routine enzymatic assays established in the central clinical chemistry laboratories of the individual study centers (Cobas 8000 c702, Advia 2400, Cobas Pro c503). LDL-C values were estimated according to the Friedewald Equation [31]. IGF-1 and IGFBP-3 were measured via electrochemiluminescence immunoassay (ECLIA) using the Cobas 8000 e801 (Roche, Switzerland), and IGFBP-2 was measured via enzyme-linked immunosorbent assay (ELISA E05, Mediagnost, Germany) at the Institut für Laboratoriumsmedizin of the LMU-Klinikum.

### Covariates

The sex, country, fasting duration, and BMI z score of the toddlers were selected a priori as potential confounders on the basis of the WHO reference population^30^. The fasting status before blood withdrawal was assessed via blood collection using the categories “less than 3 hours”, “3–6 hours” and “more than 6 hours”. The anthropometric measures were assessed at 12 and 24 months on the basis of standard operating procedures adapted from the WHO growth study [32] and the CHOP study [33]. Additionally, the study group (lower or higher protein young child formula), was considered.

### Statistical methods

The influences of visit, sex, country, fasting time and intervention group categories on the lipids and the IGF axis were analyzed via t-tests, the Stuart‒Maxwell test or ANOVA, as applicable. The relationships between IGF-1, IGFBP-3, and IGFBP-2 and the lipid parameters LDL-C, HDL-C, TC, and TG and BMI were evaluated with Pearson’s correlation coefficient. Scatter plots of the IGF-1 axis parameters and lipids are presented together with unadjusted predicted mean regression lines at 12 and 24 months. Mixed linear models with random intercepts were used to analyze the combined data of 12 and 24 months, with subjects as random effects. We tested whether estimates differed by timepoint using interaction terms and reported separate estimates by timepoint if the interaction was significant. The models were adjusted for the above named potential cofounders. As the study group did not have any significant associations with the IGF-axis or lipids, it was not considered in the models for adjustment. Significance was assumed at an error probability of 0.05; 95% confidence intervals are shown for estimates. The analyses were carried out with IBM SPSS Statistics 29, and the figures were produced via R version 4.3.1 within RStudio version 2023.09.1 + 494 ‘Desert Sunflower’.

### Ethics

Ethics approval was acquired from the ethical committees of LMU Munich, Germany (Projekt Nr. 555 − 15) and at the Institut d’Investigació Sanitaria Pere Virgili, Reus, Spain (Ref. CEIm IISPV 013/2016). The ToMI study is registered under the following number: NCT02907502. Written informed consent was obtained from parents or caregivers. All research was performed in accordance with the Declaration of Helsinki.

## Results

A total of 1624 toddlers were enrolled. Blood was drawn from 1087 children; at the 12-month visit (M ± SD: 12.7 ± 0.5 months) from 881 and at the 24-month visit (24.2 ± 0.5 months) from 775 children. The majority of the participants were from Spain (*n* = 574, 52.8%), and 585 (53.8%) of the toddlers were male. The mean BMI (kg/m^2^) of the study population was 17.0 ± 1.4 kg/m^2^ (M ± SD) at 12 months and 16.3 ± 1.3 kg/m^2^ at 24 months.

The IGF-1, IGFBP-3, LDL-C, HDL-C, and TC values were significantly higher at 24 months than at 12 months, whereas the IGFBP-2 and TG levels were significantly higher at 12 months (Table 1). Significantly more children had fasted for more than 6 h at 24 months (57%) than at 12 months of age (45%) (Table 1).

**Table 1:** Number of children, fasting status and mean serum levels of IGF-1, IGFBP-2 and IGFBP-3 as well as lipids at 12 and 24 months of age

	12 months	24 months	*p*- value^*^
Children	*n* = 881	*n* = 775	
Fasting			< 0.001
< 3 h	77 (8.7%)	62 (8.1%)	
3–6 h	409 (46.5%)	269 (35.1%)	
> 6 h	394 (44.8%)	435 (56.8%)	
IGF-1 (ng/ml)	66.3 (31.5)	84.7 (38.4)	< 0.001
IGFBP-3 (ng/ml)	2577 (630)	2691 (644)	0.001
IGFBP-2 (ng/ml)	637.6 (231.3)	499.6 (204.4)	0.001
LDL-C (mg/dl)	79.4 (23.8)	81.6 (21.8)	0.43
HDL-C (mg/dl)	39.6 (10.3)	45.2 (10.9)	0.001
TC (mg/dl)	140.4 (26.2)	143.2 (24.7)	0.11
TG (mg/dl)	115.8 (65.0)	84.8 (46.0)	0.001

*IGF-1* Insulin-like growth factor 1, *IGFBP-2, -3* Insulin-like growth factorbinding protein -2, -3, *LDL-C* Low-density lipoprotein cholesterol, *HDL-C* High-densitylipoprotein cholesterol, *TC* total cholesterol, *TG* triglycerideChildren and fasting variable aredisplayed as N (%) and blood parameters as mean (SD)

*blood parameters were compared with a paired t-test, and fasting with an asymptotic symmetry and marginal homogeneity (Stuart-Maxwell) tests for those 444 subjects with blood at both time points

The lipid levels did not significantly differ between the intervention groups at either baseline (12 months) or after the intervention (24 months) (Supplemental Table 1). BMI did not correlate with lipid parameters (all correlation coefficients < 0.08).

IGF-1, IGFBP-3, LDL-C and TC values were significantly higher in girls than in boys (Supplemental Table 2), e.g., IGF-1 + 9.8 ng/ml at 12 months and + 21.5 ng/ml at 24 months (both *p* ≤ 0.001). Additionally, these markers were significantly higher in Spain than in Germany at 12 months, e.g., IGF-1 + 14.3 ng/ml. (*p* < 0.001). IGFBP-2 levels were significantly higher in Germany than in Spain at both time points (both *p* < 0.001), e.g., IGFBP-2 + 82.7 mg/dl at 12 months (Supplemental Table 3). Only IGF-1 (*p* = 0.02) and IGFBP-2 (*p* < 0.001) were still significantly different between the two countries after considering the fasting time before blood drawn. Compared with those in Germany, more children in Spain fasted for more than 6 h (Supplemental Table 3). All parameters except of HDL-C were significantly different between different fasting categories at 12 months (all *p* < 0.001), e.g., IGFBP-3 > 6 h: 610.4 ng/ml; 3–6 h: 669.1 ng/ml; and < 3 h: 745.1 ng/ml. IGF-1 (*p* = 0.006), IGFBP-3 (*p* = 0.001), LDL-C (*p* = 0.007) and TG (*p* < 0.001) values were still significantly different between different fasting categories at 24 months, e.g., TG > 6 h: 71.8 mg/dl; 3–6 h: 101.2 mg/dl; and < 3 h: 108.7 mg/dl (Supplemental Table 4).

**Fig. 1:**
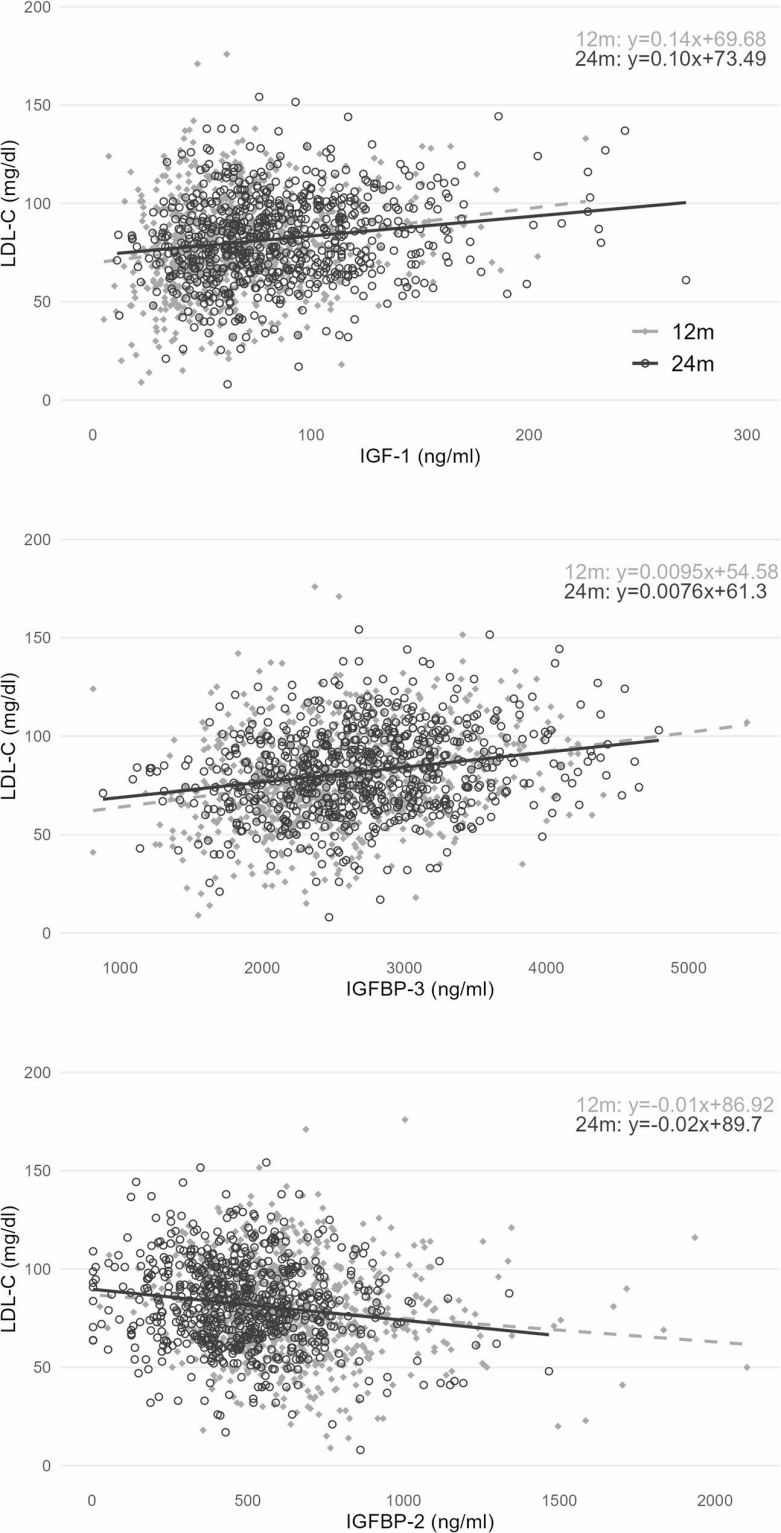
Relationship between LDL-C and the IGF-1 axis at 12 and 24 months IGF-1: Insulin-like growth factor 1; IGFBP-2,-3: Insulin-like growth factor binding protein-2, -3; LDL-C: low-density lipoprotein cholesterol. Regression terms for 12 and 24 months top right. Regression line 12 months dashed, 24 months solid line. Pearson correlation coefficient (r): IGF-1 and LDL-C 12mo 0.185***, 24 mo 0.174***; r: IGFBP-3 and LDL-C 12mo 0.250***, 24mo 0.225***; r: IGFBP-2 and LDL-C 12 mo: -0.124***, 24 mo -0.155***. Significant correlations are marked with ***<0.001, **< 0.01, * <0.05 for p-values

IGF-1 and IGFBP-3 were positively correlated with LDL-C, HDL-C and TC at 12 and 24 months of age. IGFBP-2 was negatively correlated with LDL-C, HDL-C, and TC and positively correlated with TG at both time points (Supplemental Table 5). Figure 1 illustrates the relationships of IGF-1, IGFBP-3, and IGFBP-2 with LDL-C. The figures with the lipid parameters HDL-C, TC and TG are presented in the supplementary materials (Supplemental Figs. 1–3).

Table 2 summarizes adjusted mixed model estimates of associations between IGF-1, IGFBP-3, and IGFBP-2 and lipid markers (LDL-C, HDL-C, TC, and TG). IGF-1 and IGFBP-3 were significantly positively associated, whereas IGFBP-2 was significantly negatively associated with LDL-C, HDL-C and TC. Thus, a one ng/ml change in IGF-1 was associated with a 0.08 mg/dl (95% 0.05; 0.12) increase in LDL-C or a 0.10 mg/dl (95% 0.09; 0.12) increase in HDL-C. Furthermore, IGF-1 was significantly negatively associated with TG, and IGFBP-3 (only at 12 months) and IGFBP-2 were positively associated with TG. The associations of IGF-1 with TC and of IGFBP-3 with HDL-C and TG were statistically significant at 12 and 24 months (Table 2). For example, IGF-1 was associated with a 0.205 mg/dl (95% 0.152; 0.258) increase in TC at 12 months and a 0.143 (95% 0.097; 0.189) increase at 24 months. There were no significant associations between IGFBP-3 and TG in the unadjusted model (Supplemental Table 6) or in the adjusted mixed model at 24 months (Table 2). Sex stratified mixed models revealed comparable beta coefficients (Supplemental Tables 7, 8).

**Table 2:** Mixed linear model estimates of the adjusted associations of IGF-1, IGFBP-3 and IGFBP-2 blood levels with LDL-C, HDL-C, TC and TG values at 12 and 24 months of age

	LDL-C		HDL-C		TC		TG	
	*β */95%CI]	*p* value	*β */[95%CI]	*p* value	*β* /[95%CI]	*p* value	*β* /[95%CI]	*p* value
IGF-1	0.082*** [0.049; 0.115]	< 0.001	0.105*** [0.089; 0.121]	< 0.001	12mo:0.205*** [0.152; 0.258]	0.04	-0.100* [-0.186; -0.014]	0.02
					24 mo: 0.143*** [0.097; 0.189	< 0.001		
IGFBP-3	0.007*** [0.005; 0.009]	< 0.001	12mo: 0.002*** [0.002; 0.004]	0.01	0.013*** [0.011; 0.015]	< 0.001	12mo:0.015** [0.009; 0.021]	0.02
			24mo: 0.005*** [0.004; 0.006]	< 0.001			24mo:0.005 -0.001; 0.011	0.09
IGFBP-2	-0.006** [-0.010; -0.002]	0.01	-0.011*** [-0.013; -0.008]	< 0.001	-0.012*** [-0.017; -0.007]	< 0.001	0.031*** [0.019; 0.043]	< 0.001

*IGF-1* Insulin-like growth factor 1, *IGFBP-2, -3* Insulin-like growth factor binding protein − 2, -3, *LDL-C* Low-density lipoprotein cholesterol, *HDL-C* High-density lipoprotein cholesterol, *TC* total cholesterol, *TG* triglycerides

*β* beta coefficient = effect size for one-unit changes in IGF axis parameter, *CI* confidence interval, *N* number

Mixed models adjusted for sex, country, fasting duration before blood withdrawal and BMI of the toddlers at the time of blood taking

Significant associations are marked: *p*-values ***<0.001, **< 0.01, * <0.05

If the effect of IGF axis on lipids was significantly different by time point (interaction), 12- and 24-months effect estimates were reported separately

## Discussion

We observed significant positive, but weak associations of IGF-1 and IGFBP-3 with LDL-C, HDL-C, and TC levels, as well as significant negative associations of IGFBP-2 with these lipid markers in toddlers at 12 and 24 months of age, irrespective of the intervention. The observed 0.08 mg/dl increase in LDL-C per 1 ng/ml increase of IGF-1 lies well within normal biological variability and does not necessarily indicate a clinically meaningful association.

While animal studies [11–14] make the association of the IGF-axis with circulating lipids plausible, the limited availability of studies examining IGF-1, its binding proteins, and lipid profiles in healthy children makes comparisons challenging. Most existing studies have focused on populations with conditions affecting glucose and fat metabolism, such as diabetes mellitus type 1 (DM-1) [34] or diabetes mellitus type 2 (DM-2) [35], obesity [19–21], or SGA birth [18].

GH promotes IGF-1 production and elevates insulin levels, primarily by inducing insulin resistance [36]. IGF-1, in turn, has a negative feedback loop on GH. Insulin increases liver sensitivity to GH by upregulating GH receptors and thereby elevates IGF-1 levels and lowers GH [36]. The lack of insulin by DM-1 and high insulin levels and insulin resistance by DM-2 and obesity disturb the normal balance between GH, IGF-1 and insulin [36], which explains the metabolic differences with healthy individuals.

Our findings align with a study involving SGA infants at birth that reported a positive association between IGF-1 and lipid markers, particularly TC and HDL-C levels [18]. The adjustment for birthweight and gestational age changed the associations in this reported study; thus, TG was negatively associated only after adjustment, and IGF-1 and LDL-C were no longer associated after adjustment [18]. SGA-born infants had lower IGF-1 levels than appropriate for gestational age born infants [18]. The study by Nagano [18] in SGA born infants is the only investigation of the associations of IGF-1 and lipids involving children younger than school age and therefore the closest in age to our study cohort. However, SGA birth is a characteristic that is associated with higher risk for CVD and dyslipidemia. SGA born children had elevated LDL-C, TC and TG levels, as well as reduced HDL-C levels [37]. This represents a limitation in comparison with our results.

Larger studies involving healthy participants are primarily conducted in adolescents and adults, where the influence of sex hormones on the relationship between IGF-1 and lipids is higher [38], whereas the impact of nutritional factors is relatively smaller than that in children [39]. IGF-1 was negatively associated with LDL-C in somewhat older children with short stature at a mean age of 10 years [22] as well as in children with obesity [21], while it was positively associated with LDL-C in healthy adults [15]. Consistent with our findings, positive associations of IGF-1 with HDL-C were reported in obese school-aged children [19, 20],as well as in elderly individuals [16]. In contrast, IGF-1 was negatively associated with HDL-C in healthy adults from a population-based cross-sectional analysis in Pomerania [15]. IGF-1 was positively associated with TC in adults in the same study [15], whereas it was negatively associated with TC in adults in the Framingham Heart Study [17]. No significant association between IGF-1 and lipid markers was reported in children with (pre)obesity aged 7–15 years [40]. Similarly, another study involving children and adolescents with normal glucose tolerance, impaired glucose tolerance, or DM-2 also revealed no significant associations [35].

Under GH therapy in children with GH deficiency, improvements in lipid parameters were observed in a systematic review [41]. GH therapy in children showed reduced TC, LDL-C and TG, but increased HDL-C levels [42, 43]. These results point in a different direction; however, they do not originate from toddlers.

All findings highlight the metabolic complexity and clinical inconsistency in findings concerning the relationship between IGF-1 and lipid profiles that are proposed by theoretical, biological mechanisms. Circulating IGF-1 is suggested to reduce the expression of hepatic scavenger receptor B1 on hepatocytes, which decreases the uptake of HDL-C molecules from reverse cholesterol transport into the liver, leading to a higher HDL-C concentration in the circulation [44]. On the one hand, IGF-1 and insulin share several metabolic pathways [2]. Consequently, it appears plausible that effects on lipids are similar to insulin. Insulin is known for fat storing for example as triacylglycerol in adipose tissue by influencing multiple pathways of lipid metabolism [45]. On the other hand, as reported by Clemmons et al. [10] IGF-1 may improve insulin sensitivity by suppression of insulin secretion, thereby enhancing lipolysis in adipose tissue and promoting free fatty acid use in muscle and liver. These findings seem contradictory, making it difficult to conclude about the influence of IGF-1 on lipid metabolism. It is not yet fully understood which factors determine these mechanisms; possible factors could be age, sex, genetics, weight, and diet.

Like IGF-1, IGFBP-3, the primary binding protein of IGF-1, was positively associated with lipoproteins in our analysis. Our findings align with the current literature showing positive associations of IGFBP-3 with LDL-C in adolescents with DM-1 [34] and DM-2 [35], as well as in healthy adults [15]. Additionally, positive associations were observed for IGFBP-3 and TC levels in adolescents with DM-1 [34] and DM-2 [35] as well as in those with impaired glucose tolerance [35] and in elderly individuals [16]. In adults, IGFBP-3 was negatively associated with HDL-C [15], whereas in elderly individuals, IGFBP-3 was positively associated with HDL-C [16]. In conclusion, our analysis supports and extends existing evidence that IGFBP-3, like IGF-1, is positively associated with various lipoprotein levels. Compared with IGF-1, IGFBP-3 has a more consistent and robust association with lipid profiles across diverse study populations.

In contrast to IGF-1 and IGFBP-3, IGFBP-2 was negatively associated with LDL-C, HDL-C, and TC. Our findings partly align with studies in adults [26, 27, 46], whereas very few studies have examined these associations in childhood. One study examined IGFBP-2 levels in children and adults both born SGA. They revealed a negative association between IGFBP-2 and TC and TG in the adult cohort but not in the child cohort (mean age 7.1 years) [27]. In other studies with adults, IGFBP-2 was negatively associated with LDL-C [26] and TG [26, 47, 48] but positively associated with HDL-C [46]. The negative associations between IGFBP-2 and lipoproteins may be linked to its inverse relationship with Apolipoprotein B (ApoB) [49], leading to decreases in lipoproteins.

Overall, the observed associations were largely consistent with our hypotheses; however, IGF-1 showed a positive association with LDL-C, and IGFBP-2 was negatively associated with TG, contrary to our expectations.

Our findings expand a highly heterogeneous existing evidence, particularly with regard to IGF-1. The associations of IGFBP-2 and IGFBP-3 appear more consistent. GH therapy in GH deficiency children showed lipid profile improving effects. The metabolic effects of IGF-1 in adults are highly variable. In pediatric populations, IGF-1 is more consistently positively associated with TC and HDL-C, while it tend to be negatively or not associated with LDL-C and TG. Overall, these associations seem to depend on the lipid fraction, the age group and underlying comorbidities. Definitive mechanistic explanations remain elusive. Our study extends the existing evidence to early childhood and is largely in line with previously reported findings of IGF-1 and lipids in pediatric cohorts.

### Strengths and limitations

The strengths of our study are the large sample size, the measurement at two time points in toddlerhood, the ability to adjust for the fasting duration and the assessment in a healthy population of two different countries.

The limitations of our study are that we cannot determine causality, and most effect sizes were small. In addition, we also used non-fasting lipid measurements as real fasting blood is logistically hard to obtain in this age group. While we adjusted all analysis for the fasting status, some residual confounding from current nutritional intake cannot be excluded. Adjustment generally attenuated associations. Furthermore, LDL-C values were estimated with the Friedewald equation, which tends to an underestimation of LDL-C, especially with higher triglycerides. However, our TG levels were relatively low (mean 101.2 mg/dL, SD 58.9) in respect to the acceptable range for its application [50]. The further follow-up results of the ToMI study will be helpful for observing the relationship between the IGF axis and lipids until 6 years of life.

## Conclusion

The IGF axis is weakly associated with circulating lipid levels. IGF-1 and IGFBP-3 were positively associated with LDL-C, HDL-C and TC, while IGFBP-2 was negatively associated with lipoprotein levels. However, the effect sizes were generally modest. Overall, associations of the IGF axis with lipid profiles seem to depend on the lipid fraction, the age group and underlying comorbidities.

## Supplementary Information

Supplementary Material 1.

## Data Availability

Some or all datasets prepared and analyzed during this study are not publicly available. They are available from the corresponding author on reasonable request.

## Abbreviations

ApoB: Apolipoprotein B
BMI: Body mass index
ECLIA: Electrochemiluminescence-immunoassay
ELISA: Enzyme-linked immunosorbent assay
GH: Growth hormone
HDL-C: High-density lipoprotein cholesterol
IGF-1: Insulin-like growth factor 1
IGFBP-2: Insulin-like growth factor binding protein 2
IGFBP-3: Insulin-like growth factor binding protein 3
LDL-C: Low-density lipoprotein cholesterol
SD: Standard deviation
SGA: Small for gestational age
TC: Total cholesterol
TG: Triglycerides
ToMI: Toddler Milk Intervention trial
