# Supplementary material for: Associations of insulin-like growth factor 1 and IGF binding proteins 2 and 3 with lipids in toddlers
Source: Lipids Health Dis. 2026 Feb 3;25:68. doi: 10.1186/s12944-026-02872-y (PMC12934086; doi:10.1186/s12944-026-02872-y)
Supplement: Supplementary file 1 — Supplementary Material 1. [file 12944_2026_2872_MOESM1_ESM.docx]

Supplements

|  | 12 months | | | 24 months | | |
| --- | --- | --- | --- | --- | --- | --- |
|  | higher protein | lower protein | P values | higher protein | lower protein | P values |
| **LDL-C** (mg/dl) | 80.0 (24.0) | 78.9 (23.5) | 0.528 | 82.7 (22.6) | 80.7 (20.9) | 0.209 |
| **HDL-C** (mg/dl) | 39.7 (10.3) | 39.5 (10.4) | 0.770 | 44.6 (11.4) | 45.6 (10.3) | 0.222 |
| **TC** (mg/dl) | 140.8 (26.2) | 140.0 (26.1) | 0.670 | 143.7 (25.5) | 142.8 (23.9) | 0.606 |
| **TG** (mg/dl) | 115.4 (61.0) | 116.1 (68.9) | 0.855 | 84.6 (47.1) | 84.8 (45.1) | 0.955 |
| *LDL-C Low-density lipoprotein cholesterol; HDL-C High-density lipoprotein cholesterol; TC total cholesterol; TG triglycerides*  *Data are displayed as mean (SD)*  *Protein* *groups were compared with unpaired t-tests* | | | |  |  |  |

**Supplemental Table 1** Comparison of lipid levels between protein groups

**Supplemental Table 2** Sex differences of IGF axis and lipids

|  | 12 months | | | 24 months | | | |
| --- | --- | --- | --- | --- | --- | --- | --- |
|  | **Female**  **n=379** | **Male**  **n=453** | **P value** | **Female**  **n=346** | **Male**  **n=399** | **P value** | |
| **IGF-1** | 71.6 (34.0) | 61.8 (28.6) | <0.001 | 96.2 (39.3) | 74.7 (34.6) | <0.001 | |
| **IGFBP-3** | 2.68 (0.65) | 2.49 (0.60) | <0.001 | 2.85 (0.60) | 2.55 (0.65) | <0.001 | |
| **IGFBP-2** | 642.9 (249.0) | 657.1 (251.1) | 0.416 | 481.3 (211.5) | 501.0 (210.6) | 0.205 | |
| **LDL-C** | 83.3 (23.6) | 76.1 (23.5) | <0.001 | 84.3 (22.9) | 79.3 (20.5) | 0.002 | |
| **HDL-C** | 39.2 (10.0) | 40.0 (10.6) | 0.305 | 44.6 (10.1) | 44.6 (10.9) | 0.160 | |
| **TC** | 144.6 (26.5) | 136.9 (25.4) | <0.001 | 145.9 (25.8) | 140.9 (23.4) | 0.006 | |
| **TG** | 119.2 (63.4) | 112.8 (66.2) | 0.159 | 87.8 (41.3) | 82.1 (49.7) | 0.096 | |
| *IGF-1 Insulin-like growth factor 1; IGFBP-2, -3 Insulin-like growth factor binding protein -2, -3; LDL-C Low-density lipoprotein cholesterol; HDL-C High-density lipoprotein cholesterol; TC total cholesterol; TG triglycerides, n number, IGF axis values in ng/ml, lipids in mg/dl, Data are displayed as mean (SD) Sex was compared with unpaired t-tests* | | | | | | |  |

**Supplemental Table 3** Country differences of IGF-axis and lipids

|  | 12 months | | | 24 months | | | |
| --- | --- | --- | --- | --- | --- | --- | --- |
|  | **Germany**  n=391 | **Spain**  n=483 | **P value** | **Germany**  n=363 | **Spain**  n=406 | **P value** | |
| **IGF-1** | 58.5 (24.6) | 72.6 (34.9) | <0.001 | 78.2 (31.4) | 93.7 (42.9) | <0.001 | |
| **IGFBP-3** | 2.39 (0.55) | 2.73 (0.65) | <0.001 | 2.65 (0.60) | 2.73 (0.68) | 0.125 | |
| **IGFBP-2** | 694.4 (254.5) | 611.7 (239.7) | <0.001 | 571.5 (184.0) | 416.2 (207.6) | <0.001 | |
| **LDL-C** | 72.8 (22.6) | 84.1 (23.5) | <0.001 | 80.3 (21.2) | 81.9 (22.2) | 0.703 | |
| **HDL-C** | 39.3 (10.4) | 39.9 (10.3) | 0.428 | 45.2 (10.2) | 45.1 (10.3) | 0.963 | |
| **TC** | 135.5 (24.9) | 143.9 (26.6) | <0.001 | 144.6 (24.1) | 142.1 (25.1) | 0.173 | |
| **TG** | 136.3 (80.8) | 110.1 (45.5) | <0.001 | 96.0 (50.4) | 75.6 (39.9) | <0.001 | |
| *IGF-1 Insulin-like growth factor 1; IGFBP-2,-3 Insulin-like growth factor binding protein -2, -3, LDL-C low density*  *lipoprotein cholesterol, HDL-C High-density lipoprotein cholesterol; TC total cholesterol; TG triglycerides, n number*  *IGF axis values in ng/ml, lipids in mg/dl*  *Data are displayed as mean (SD) Country was compared with unpaired t-tests* | | | | | | |  |

**Supplemental Table 4** Fasting differences of IGF-axis and lipids

|  |  | **12 months** | | | **24 months** | | |
| --- | --- | --- | --- | --- | --- | --- | --- |
|  | **fasting** | **n** | **M(SD)** | **P value** | **n** | **M(SD)** | **P value** |
| **IGF-1** | >6 hours | 359 | 73.68 (35.37) | <0.001 | 403 | 88.55 (41.60) | 0.006 |
|  | 3-6 hours | 381 | 60.44 (26.95) |  | 255 | 80.91 (35.17) |  |
|  | <3 hours | 70 | 60.01 (25.92) |  | 59 | 75.29 (24.06) |  |
| **IGFBP-2** | >6 hours | 361 | 2.77 (0.65) | <0.001 | 404 | 2.73 (0.66) | 0.065 |
|  | 3-6 hours | 382 | 2.44 (0.57) |  | 255 | 2.66 (0.64) |  |
|  | <3 hours | 70 | 2.39 (0.58) |  | 59 | 2.55 (0.54) |  |
| **IGFBP-3** | >6 hours | 355 | 610.4 (241.3) | <0.001 | 408 | 440.8 (218.3) | <0.001 |
|  | 3-6 hours | 397 | 669.1 (245.3) |  | 267 | 550.0 (179.4) |  |
|  | <3 hours | 77 | 745.1 (279.5) |  | 61 | 576.8 (201.4) |  |
| **LDL-C** | >6 hours | 386 | 86.0 (23.3) | <0.001 | 423 | 83.3 (22.4) | 0.007 |
|  | 3-6 hours | 371 | 73.5 (22.5) |  | 246 | 80.2 (20.8) |  |
|  | <3 hours | 63 | 73.4 (24.1) |  | 57 | 74.2 (20.0) |  |
| **HDL-C** | >6 hours | 374 | 40.1 (10.3) | 0.449 | 423 | 45.4 (11.3) | 0.318 |
|  | 3-6 hours | 373 | 39.2 (10.4) |  | 246 | 45.1 (10.4) |  |
|  | <3 hours | 65 | 39.2 (9.8) |  | 57 | 43.1 (10.3) |  |
| **TC** | >6 hours | 741 | 144.6 (25.6) | <0.001 | 424 | 142.9 (25.5) | 0.116 |
|  | 3-6 hours | 131 | 134.2 (25.9) |  | 246 | 140.5 (25.1) |  |
|  | <3 hours | 14 | 138.2 (25.5) |  | 57 | 137.5 (21.0) |  |
| **TG** | >6 hours | 741 | 94.2 (61.8) | <0.001 | 424 | 71.8 (32.3) | <0.001 |
|  | 3-6 hours | 131 | 132.7 (72.2) |  | 246 | 101.2 (45.3) |  |
|  | <3 hours | 14 | 146.2 (51.9) |  | 57 | 108.7 (45.9) |  |
| *IGF-1 Insulin-like growth factor 1; IGFBP-2, -3 Insulin-like growth factor binding protein -2, -3; LDL-C Low-density lipoprotein cholesterol; HDL-C High-density lipoprotein cholesterol; TC total cholesterol;TG triglycerides, n number*  *IGF axis values in ng/ml, lipids in mg/dl*  *Data are displayed as mean (SD), fasting categories were compared with ANOVA* | | | | | | | |

| ***Supplemental Table 5*** *Correlations of IGF-1, IGFBP-2 and IGFBP-3, with the blood markers LDL-C, HDL-C, TC and TG at 12 and 24 months of age* | | | | | | | | | |
| --- | --- | --- | --- | --- | --- | --- | --- | --- | --- |
|  | | **LDL-C** | | **HDL-C** | | **TC** | | **TG** | |
|  |  | r | n | r | n | r | n | r | n |
| 12 mo | IGF-1 | 0.185*** | 780 | 0.209*** | 774 | 0.249*** | 787 | -0.39 | 787 |
|  | IGFBP-2 | -0.124** | 771 | -0.139*** | 767 | -0.145*** | 778 | 0.094** | 778 |
|  | IGFBP-3 | 0.250*** | 783 | 0.118*** | 776 | 0.307*** | 790 | 0.058 | 790 |
| 24 mo | IGF-1 | 0.174*** | 702 | 0.177*** | 702 | 0.227*** | 703 | -0.019 | 703 |
|  | IGFBP-2 | 0.155*** | 705 | -0.143*** | 705 | -0.147*** | 706 | 0.165*** | 706 |
|  | IGFBP-3 | 0.225*** | 703 | 0.225*** | 703 | 0.313*** | 704 | 0.039 | 704 |
| *IGF-1 Insulin-like growth factor 1; IGFBP-2, -3 Insulin-like growth factor binding protein -2, -3; LDL-C Low-density lipoprotein cholesterol; HDL-C High-density lipoprotein cholesterol; TC total cholesterol; TG triglycerides*  *r correlation coefficient, n number*  *Significant correlations are marked with ***<0.001, **< 0.01, * <0.05 for p-values* | | | | | | | | | |

| ***Supplemental Table 6*** *Mixed linear model estimates of the unadjusted associations of IGF-1, IGFBP-2 and IGFBP-3 serum levels with LDL-C, HDL-C, TC and TG values at 12 and 24 months of age* | | | | | | | | | |
| --- | --- | --- | --- | --- | --- | --- | --- | --- | --- |
|  | **LDL-C** | | **HDL-C** | | **TC** | | **TG** | | |
|  | β/95%CI] | P value | β /[95%CI] | P value | β /[95%CI] | P value | β /[95%CI] | P value |  |
| **IGF-1**  **IGFBP-3**  **IGFBP-2** | 0.121***  [0.089; 0.153] | <0.001 | 0.095***  [0.080; 0.110] | <0.001 | 0.187***  [0.152; 0.222] | <0.001 | -0.180***  [-0.265; -0.095] | <0.001 |  |
|  | 0.009***  [0.007; 0.011] | <0.001 | 0.004***  [0.003.; 0.005] | <0.001 | 0.014***  [0.012; 0.016] | <0.001 | 0.002  [-0.003.; 0.007] | 0.379 |  |
|  |  |  |  |  |  |  |  |  |  |
|  | -0.011***  [-0.015; -0.006] | <0.001 | -0.010***  [-0.012; -0.008] | <0.001 | -0.014***  [-0.019; -0.010] | <0.001 | 0.046***  [0.034; 0.058] | <0.001 |  |
| *IGF-1 Insulin-like growth factor 1; IGFBP-2, -3 Insulin-like growth factor binding protein -2, -3; LDL-C Low-density lipoprotein cholesterol; HDL-C High-density lipoprotein cholesterol; TC total cholesterol; TG triglycerides*  *β beta coefficient=effect size for one-unit changes in IGF axis parameter; CI: confidence interval*  *Significant associations are marked with ***<0.001, **< 0.01, * <0.05 for p-values* | | | | | | | | | |

| **Supplemental Table 7** Sex stratified mixed linear model estimates of the adjusted associations of IGF-1, IGFBP-3 and IGFBP-2 serum levels with LDL-C, HDL-C, TC and TG values at 12 and 24 months of age  **Sex: Female** | | | | | | | | |
| --- | --- | --- | --- | --- | --- | --- | --- | --- |
|  | **LDL-C** | | **HDL-C** | | **TC** | | **TG** | |
|  | β/95%CI] | p value | β /[95%CI] | p value | β /[95%CI] | p value | β /[95%CI] | p value |
| **IGF-1** | 0.067***  [0.022; 0.113] | 0.01 | 0.103***  [0.082; 0.124] | <0.001 | 12mo:0.195  [0.045; 0.344] | 0.28  <0.001 | -0.133*  [-0.243; -0.023] | 0.02 |
|  |  |  |  |  | 24 mo: 0.149***  [0.083; 0.214] |  |  |  |
| **IGFBP-3** | 0.007***  [0.004; 0.01] | 0.01 | 12mo: 0.001  [-0.001; 0.007] | 0.41  <0.001 | 0.013***  [0.010; 0.016] | <0.001 | 12mo:0.014  [-0.029; 0.058] | 0.61  0.23 |
|  |  |  | 24mo: 0.002***  [0.004; 0.006] |  |  |  | 24mo:0.006  -0.004; 0.015 |  |
| **IGFBP-2** | -0.005  [-0.011; 0.002] | 0.16 | -0.011***  [-0.015; -0.008] | <0.001 | -0.011**  [-0.019; -0.004] | <0.01 | 0.032***  [0.015; 0.049] | <0.001 |
| *IGF-1 Insulin-like growth factor 1; IGFBP-2, -3 Insulin-like growth factor binding protein -2, -3; LDL-C Low-density lipoprotein cholesterol; HDL-C High-density lipoprotein cholesterol; TC total cholesterol; TG triglycerides*  *β beta coefficient=effect size for one-unit changes in IGF axis parameter; CI: confidence interval, N number*  *Mixed models adjusted for sex, country, fasting duration before blood withdrawal and BMI of the toddlers at the time of blood taking*  *Significant associations are marked: p-values ***<0.001, **< 0.01, * <0.05*  *If the effect of IGF axis on lipids was significantly different by time point (interaction), 12- and 24-months effect estimates were reported separately* | | | | | | | | |

| **Supplemental Table 8** Sex stratified mixed linear model estimates of the adjusted associations of IGF-1, IGFBP-3 and IGFBP-2 serum levels with LDL-C, HDL-C, TC and TG values at 12 and 24 months of age  **Sex: Male** | | | | | | | | |
| --- | --- | --- | --- | --- | --- | --- | --- | --- |
|  | **LDL-C** | | **HDL-C** | | **TC** | | **TG** | |
|  | β/95%CI] | p value | β /[95%CI] | p value | β /[95%CI] | p value | β /[95%CI] | p value |
| **IGF-1** | 0.099***  [0.049; 0.148] | <0.001 | 0.108***  [0.084; 0.133] | <0.001 | 12mo:0.200  [0.045; 0.366] | 0.38  <0.001 | -0.071  [-0.204; -0.062] | 0.30 |
|  |  |  |  |  | 24 mo: 0.161***  [0.093; 0.229] |  |  |  |
| **IGFBP-3** | 0.007***  [0.004; 0.009] | <0.001 | 12mo: 0.003***  [-0.001; 0.006] | 0.01  <0.001 | 0.013***  [0.010; 0.016] | <0.001 | 12mo:0.016  [-0.004; 0.035] | 0.08  0.21 |
|  |  |  | 24mo: 0.005***  [0.004; 0.007] |  |  |  | 24mo:0.005  -0.003; 0.014 |  |
| **IGFBP-2** | -0.007**  [-0.013; -0.002] | 0.01 | -0.010***  [-0.013; -0.007] | <0.001 | -0.012***  [-0.019; -0.006] | <0.001 | 0.031***  [0.014; 0.048] | <0.001 |
| *IGF-1 Insulin-like growth factor 1; IGFBP-2, -3 Insulin-like growth factor binding protein -2, -3; LDL-C Low-density lipoprotein cholesterol; HDL-C High-density lipoprotein cholesterol; TC total cholesterol; TG triglycerides*  *β beta coefficient=effect size for one-unit changes in IGF axis parameter; CI: confidence interval, N number*  *Mixed models adjusted for sex, country, fasting duration before blood withdrawal and BMI of the toddlers at the time of blood taking*  *Significant associations are marked: p-values ***<0.001, **< 0.01, * <0.05*  *If the effect of IGF axis on lipids was significantly different by time point (interaction), 12- and 24-months effect estimates were reported separately* | | | | | | | | |

***
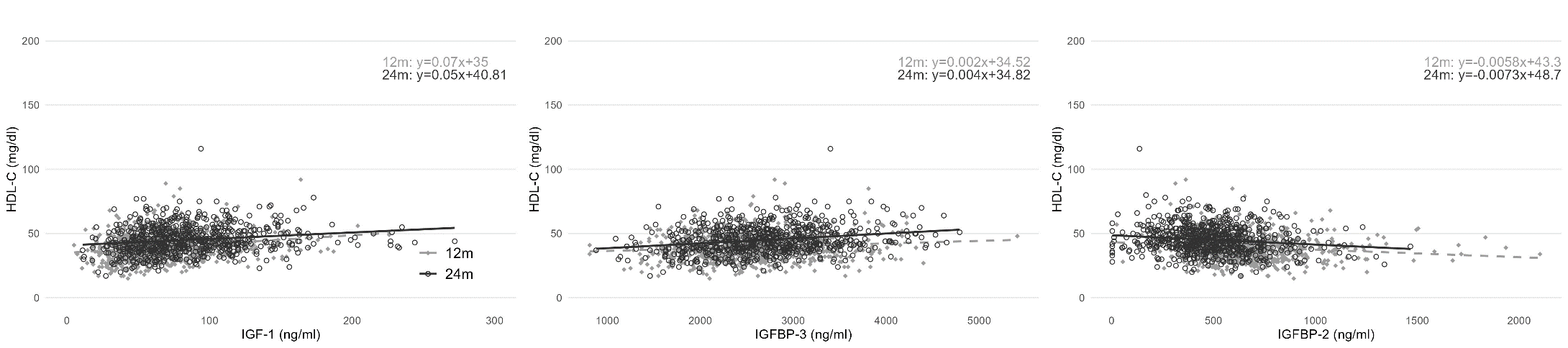
***

***Supplemental Figure 1:*** *Relationship between HDL-C and IGF-1 axis parameters at 12 and 24 months*

IGF-1 Insulin-like growth factor 1;IGFBP-2,-3 Insulin-like growth factor-2, -3; HDL-C high-density lipoprotein cholesterol

Regression terms for 12 and 24 months top right

Regression line 12 months dashed, 24 months solid line

Pearson correlation coefficient (r): IGF-1 and HDL-C 12mo 0.209***, 24 mo 0.177***; r: IGFBP-3 and HDL-C 12mo 0.118***, 24mo 0.225***;

r: IGFBP-2 and HDL-C 12 mo: -0.139***, 24 mo -0.143***

Significant correlations are marked with ***<0.001, **< 0.01, * <0.05 for p-values


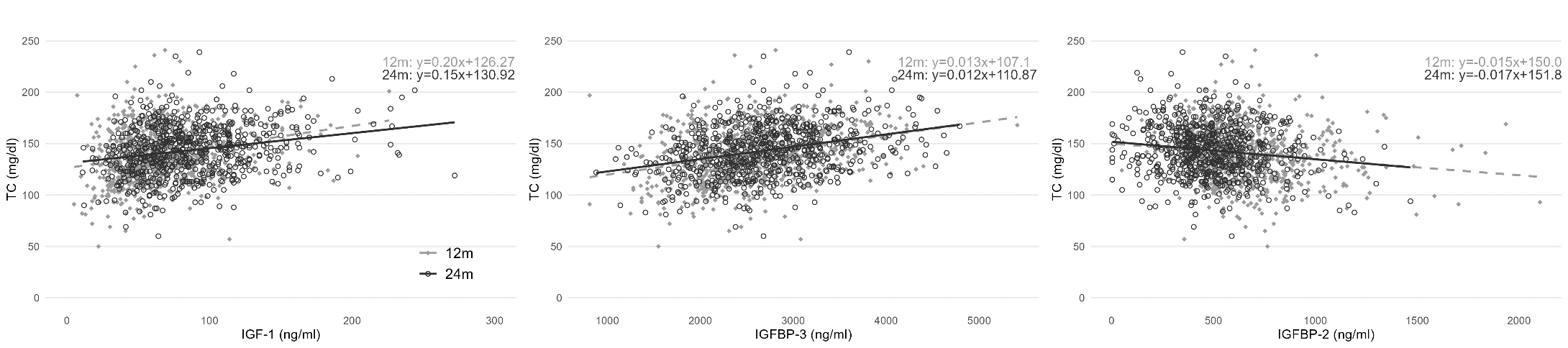


***Supplemental Figure 2:*** *Relationship between TC and IGF-1 axis parameters at 12 and 24 months*

IGF-1 Insulin-like growth factor 1;IGFBP-2,-3 Insulin-like growth factor-2, -3; TC total cholesterol

Regression terms for 12 and 24 months top right

Regression line 12 months dashed, 24 months solid line

Pearson correlation coefficient (r): IGF-1 and TC 12mo 0.249***, 24 mo 0.227***; r: IGFBP-3 and TC 12mo 0.307***, 24mo 0.313***;

r: IGFBP-2 and TC 12 mo: -0.145***, 24 mo -0.147***

Significant correlations are marked with ***<0.001, **< 0.01, * <0.05 for p-values


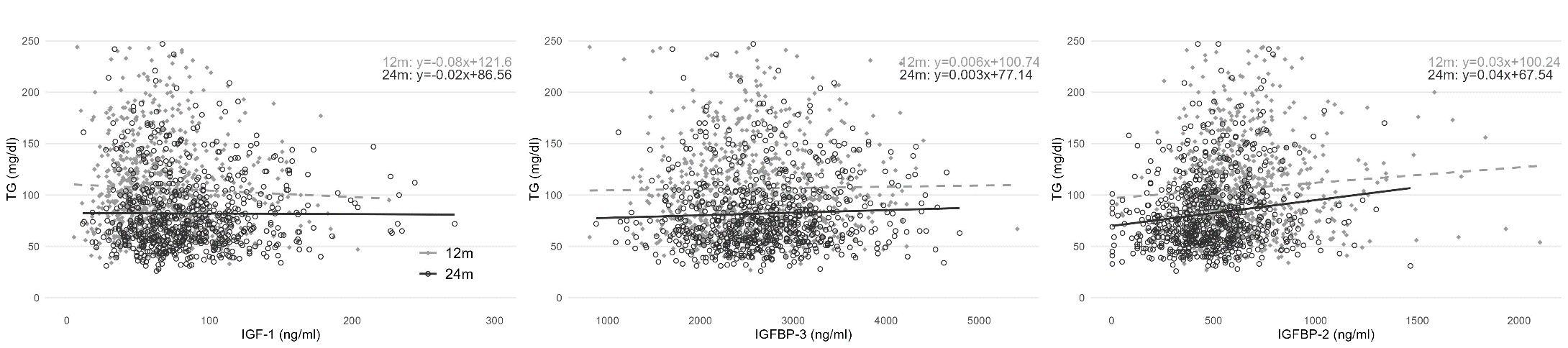


***Supplemental Figure 3:*** *Relationship between TG and IGF-1 axis parameters at 12 and 24 months*

IGF-1 Insulin-like growth factor 1;IGFBP-2,-3 Insulin-like growth factor-2, -3; TG triglycerides

Regression terms for 12 and 24 months top right

Regression line 12 months dashed, 24 months solid line

Pearson correlation coefficient (r): IGF-1 and TG 12mo -0.39, 24 mo -0.019; r: IGFBP-3 and TG 12mo 0.058, 24mo 0.039;

r: IGFBP-2 and TG 12 mo: 0.094**, 24 mo 0.165***

Significant correlations are marked with ***<0.001, **< 0.01, *<0.05 for p-values
